# Supplementary material for: Co‐translational insertion and topogenesis of bacterial membrane proteins monitored in real time
Source: EMBO J. 2020 Apr 20;39(15):e104054. doi: 10.15252/embj.2019104054 (PMC7396858; doi:10.15252/embj.2019104054)
Supplement: Supplementary file 1 — Appendix [file EMBJ-39-e104054-s001.pdf]

Appendix to:

Co-translational insertion and topogenesis of bacterial membrane proteins monitored in real time

Evan Mercier, Wolfgang Wintermeyer, Marina V. Rodnina*

Content

Appendix Figure S1:	Model of the RNC–SecY complex
Appendix Figure S2:	Co-translational membrane insertion of LepB monitored by FRET
Appendix Figure S3:	Translation time courses for LepB mRNA constructs
Appendix Figure S4:	Co-translational membrane insertion of EmrD monitored by FRET
Appendix Figure S5:	Translation time courses for EmrD and EmrD(–) mRNA constructs
Appendix Figure S6:	Translation time courses of EmrD and EmrD(–)
Appendix Figure S7:	Validation of EmrD(–) fitting
Appendix Figure S8:	Co-translational protection of nascent chains against PK digestion by the ribosome and SecYEG
Appendix Table S1:	LepB translation and FRET kinetics evaluated by exponential fitting
Appendix Table S2:	EmrD translation and FRET kinetics evaluated by exponential fitting
Appendix Table S3:	EmrD(–) translation and FRET kinetics evaluated by exponential fitting

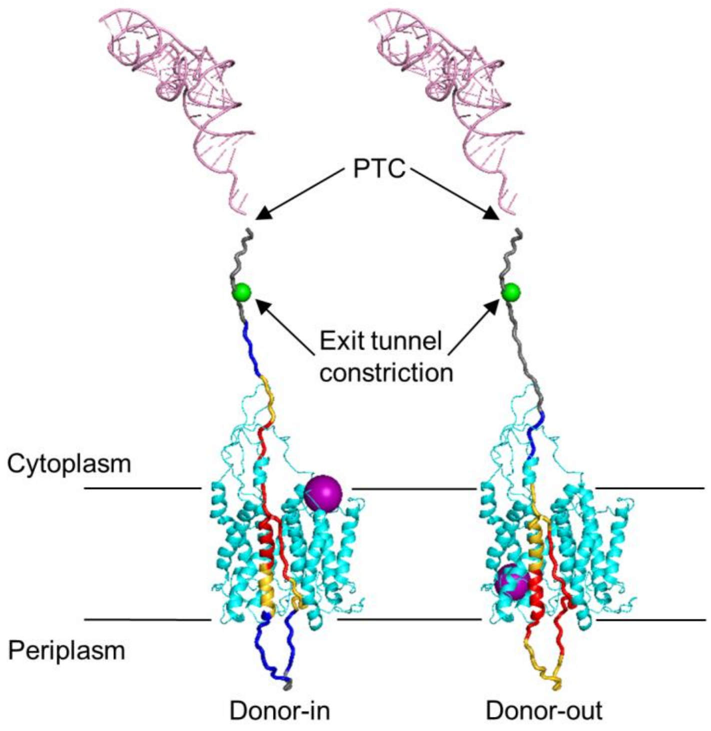

Appendix Fig S1 - Model of the RNC–SecY complex.

The P-site tRNA is shown in pink, and the peptidyl transferase center (PTC) is indicated. The nascent chain is shown as a ribbon and colored according to the expected approximate FRET efficiency as follows: < 10% gray, 10%-50% blue, 50%-90% yellow, >90% red. The constriction formed by ribosomal proteins L4 and L22 is indicated by a green sphere. SecY is shown in cyan and the position of the donor fluorophore is shown as a purple sphere for donor-in (position 111, left) and donor-out (position 212, right) constructs. The model is based on the cryo-EM structure of an RNC–SecYEG complex PDBID:4V6M (Frauenfeld et al., 2011). Related to Fig 1.

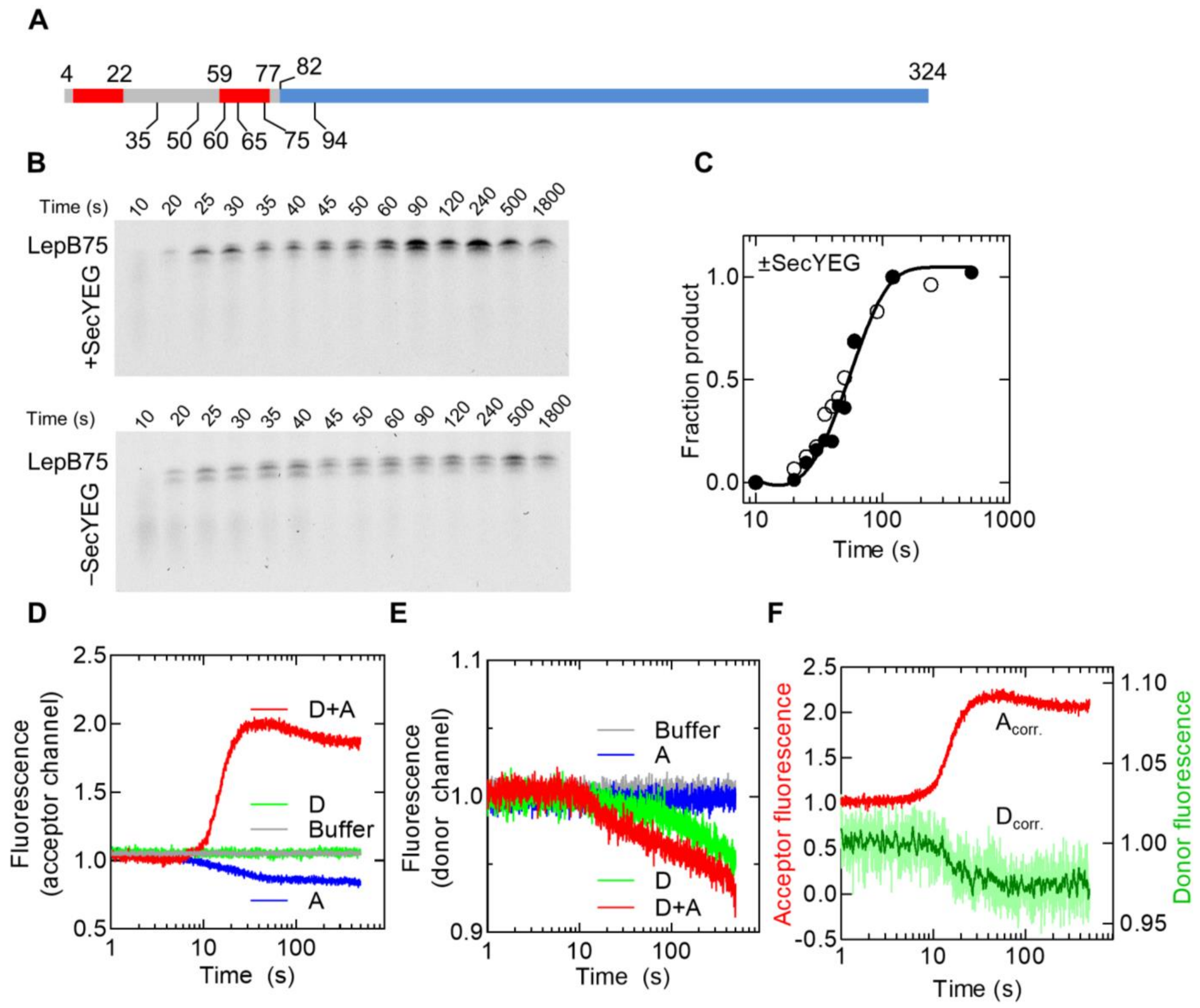

Appendix Fig S2 - Co-translational membrane insertion of LepB monitored by FRET.

A Schematic of LepB with TMs colored red and the periplasmic domain colored blue. Amino acids at the start and end of each structural element are indicated on top of the schematic, while nascent chain lengths investigated in this study are indicated below.

B Time courses of LepB75 translation were performed with SecYEG (0.5 µM) (upper panel) or without SecYEG (lower panel).

C Quantification of the time courses in panel B.

D (B-D) FRET controls for co-translational LepB75 insertion into SecYEG. The nascent chain was labeled with Atto655 acceptor (A) at the N-terminus, SecY was labeled with Atto488 at position 111 as donor (D).

E Fluorescence changes observed in the acceptor channel. D, donor only; A, acceptor only; D+A, donor plus acceptor; buffer only.

F Fluorescence changes observed in the donor channel. See panel D. Corrected fluorescence changes of donor and acceptor ($F_{corr}=F_{D+A}-F_{A\text{-}only}-F_{D\text{-}nly}+F_{buffer}$). Related to Figure 1.

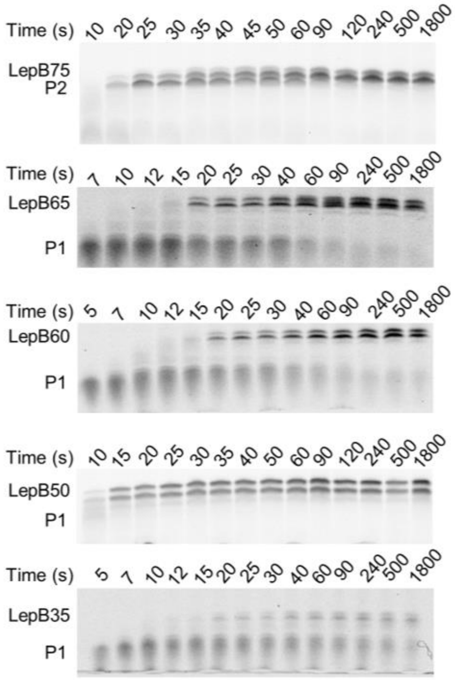

Appendix Fig S3 - Translation time courses for LepB mRNA constructs.

Peptides that accumulate due to translational pausing are indicated as P1 and P2. Related to Figure 2

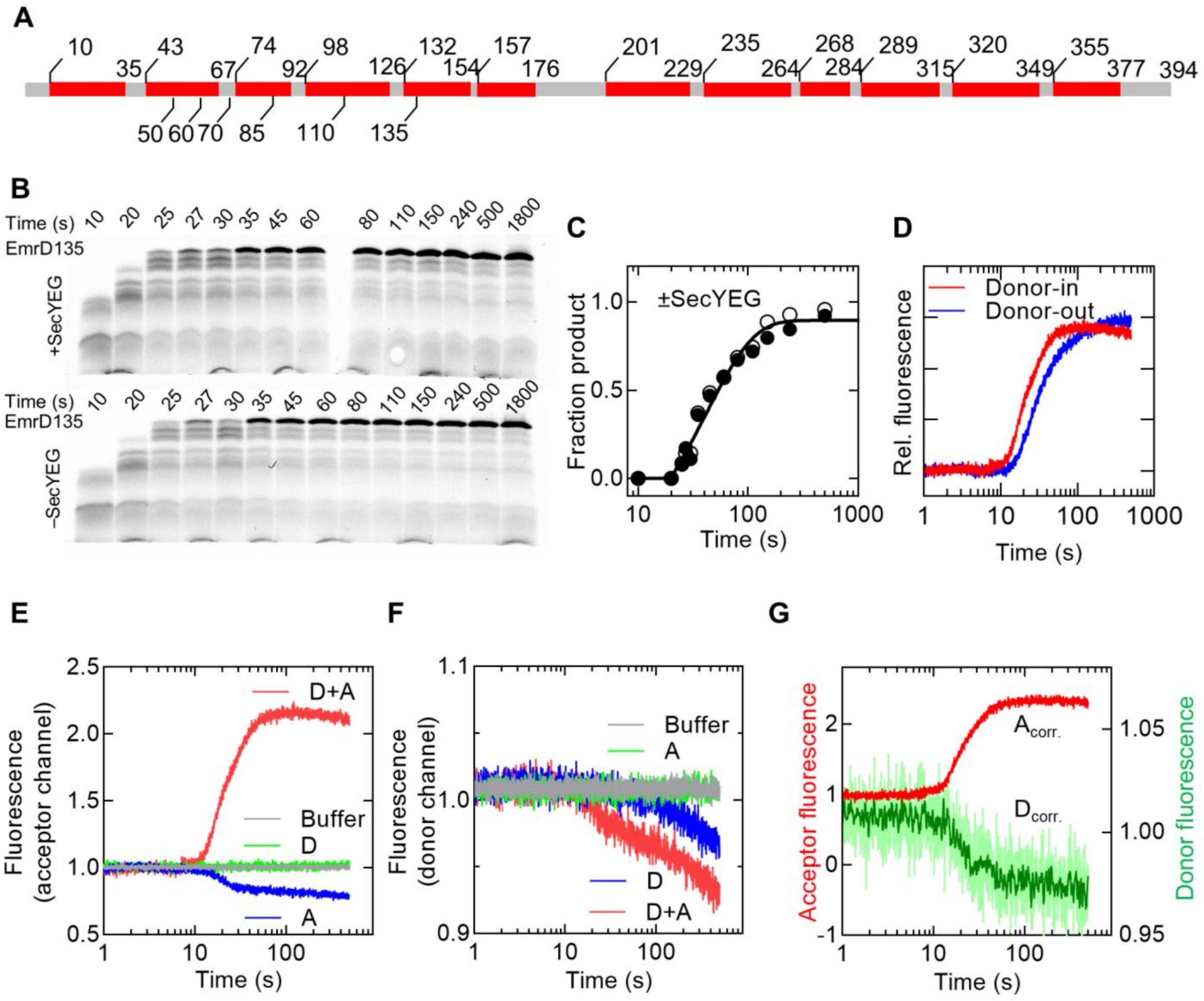

Appendix Fig S4 - Co-translational membrane insertion of EmrD monitored by FRET.

A Schematic of EmrD with TMs colored red and loops colored gray. Amino acids at the start and end of each structural element are indicated on top of the schematic, while nascent chain lengths investigated in this study are indicated below.

B Time courses of EmrD135 translation performed with (upper panel) and without (lower panel) SecYEG (0.5 µM).

C Time courses of EmrD translation with and without SecYEG. Quantification of the data in panel B.

D Time-dependent acceptor fluorescence changes during co-translational insertion of EmrD135 into donor-labeled translocons.

E Fluorescence change during EmrD translation observed in the acceptor channel. D, donor only; A, acceptor only; D+A, donor plus acceptor; buffer only.

F Fluorescence change during EmrD translation observed in the donor channel.

G Corrected donor- and acceptor-fluorescence ($F_{corr.} = F_{D+A} – F_{A\text{-}only} – F_{D\text{-}only} + F_{buffer}$). Related to Figure 3.

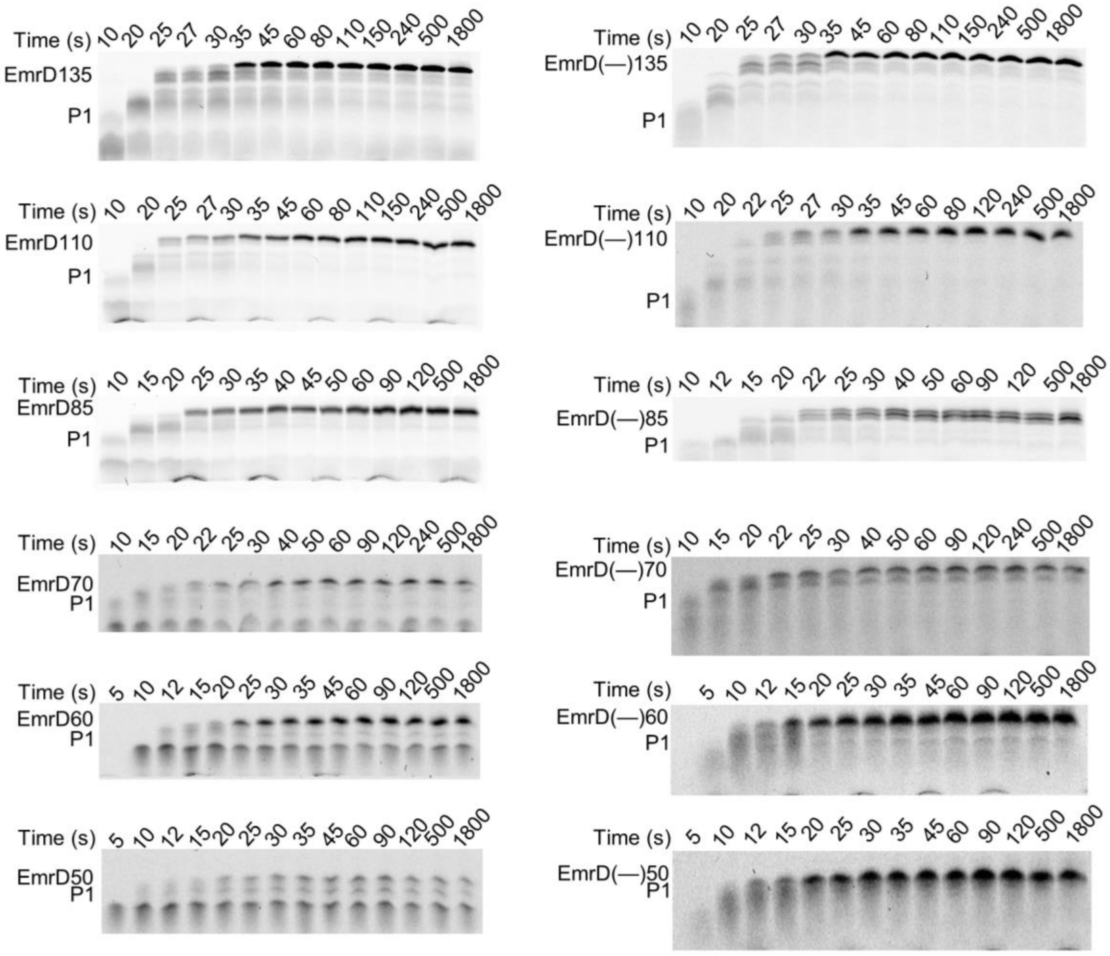

Appendix Fig S5 - Translation time courses for EmrD and EmrD(–) mRNA constructs.

Peptides that accumulate due to translational pausing are indicated as P1. Related to Figure 3. Translation time courses for EmrD 135 and EmrD(–) 135 (top left and top right, respectively) are the same as shown in Fig 3A and B, respectively.

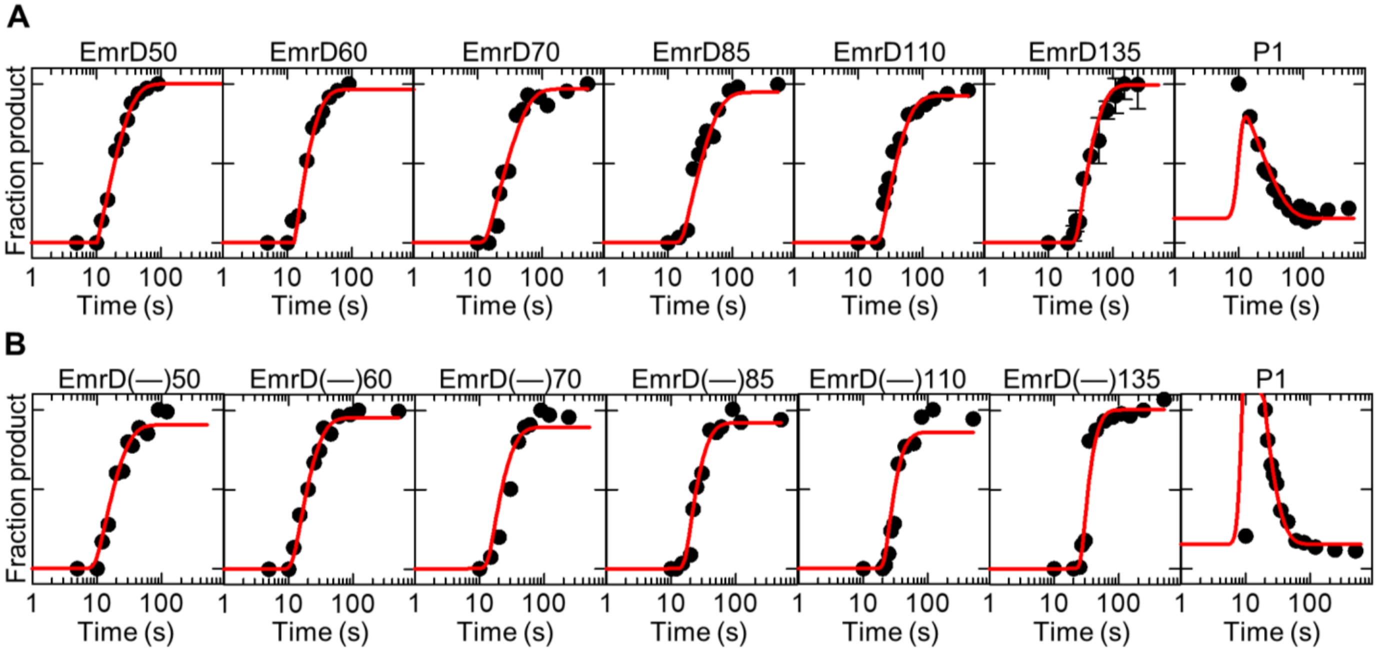

Appendix Fig S6 - Translation time courses.

A EmrD mRNA constructs.

B EmrD(–) mRNA constructs.

Experimental points are depicted as filled circles (from Appendix Fig S5) along with the resulting global fits (red lines). Related to Figure 3.

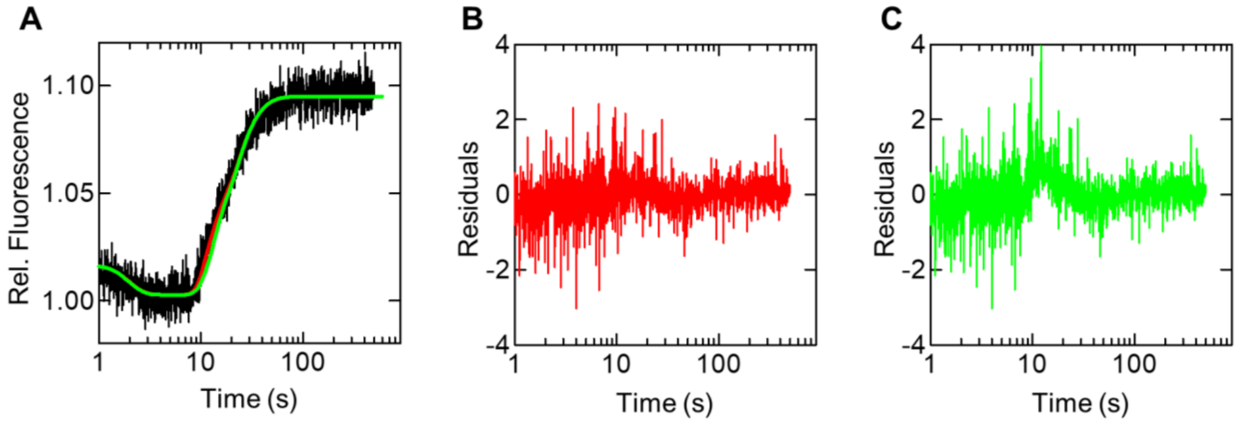

Appendix Fig S7 - Validation of EmrD(–) fitting.

A FRET stopped-flow traces for EmrD(–)135 insertion into donor-in labeled SecYEG. Fits resulting from models with and without a high-FRET intermediate are shown as red and green lines, respectively.

B Residuals calculated for fits with the high-FRET intermediate.

C Residuals calculate for fits without the high-FRET intermediate. Related to Figure 3.

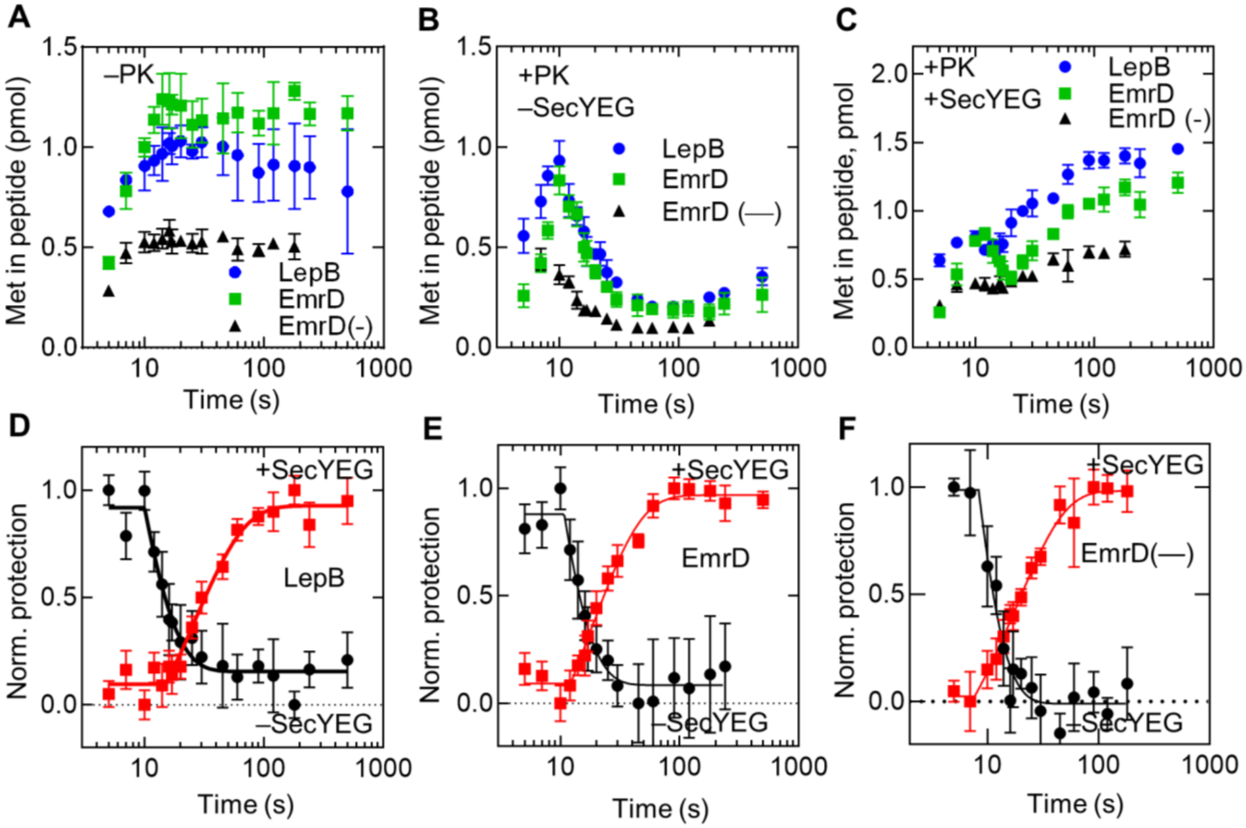

Appendix Fig S8 - Co-translational protection of nascent chains against PK digestion by the ribosome and SecYEG.

A ^{3}H-Met-containing peptides were precipitated with TCA after the indicated translation time in the absence of PK.

B As in A, but in the presence of PK.

C As in A and B, but in the presence of additionally SecYEG.

D Co-translational digestion of LepB nascent chains by PK in the absence (black symbols) or presence (red symbols) of SecYEG. The relative protection was calculated as follows: Protection(–SecYEG) = 1 + Met(+PK–SecYEG) – Met(–PK) and Protection(+SecYEG) = Met(+PK+SecYEG) – Met(+PK–SecYEG). Lines were obtained by fitting to delay-exponential functions.

E Same as D for EmrD

F Same as (D) for EmrD(–). Related to Figure 4.

Appendix Table S1 - LepB translation and FRET kinetics evaluated by exponential fitting.

mRNA (codons)	τ_{av}[a] (s)	k_{av}[b] (aa/s)	τ_1[c] (s)	τ_2[c] (s)	τ_{post}[c]. (s)
35	26 ± 6	1.4 ± 0.3	-	-	300 ± 100[d]
50	21 ± 8	2.4 ± 0.6	23 ± 1	-	-
60	40 ± 10	1.5 ± 0.2	18 ± 1	-	-
65	34 ± 7	1.9 ± 0.2	16 ± 1	-	-
75	64 ± 13	1.2 ± 0.1	15 ± 1	50 ± 2	-
94	54 ± 6	1.7 ± 0.1	15 ± 1	64 ± 4	-

Appendix Table S2 - EmrD translation and FRET kinetics evaluated by exponential fitting.

mRNA (codons)	τ_{av}[a] (s)	k_{av}[b] (aa/s)	τ_1[c] (s)	τ_2[c] (s)	τ_{post}[c]. (s)
50	23 ± 3	2.2 ± 0.3	36 ± 5	-	-
60	23 ± 4	2.6 ± 0.5	30 ± 1	-	-
70	31 ± 7	2.2 ± 0.5	30 ± 1	-	-
85	37 ± 9	2.3 ± 0.6	25 ± 1	-	310 ± 110
110	38 ± 6	2.9 ± 0.4	34 ± 1	-	180 ± 90[d]
135	54 ± 10	2.5 ± 0.5	36 ± 1	-	-

Appendix Table S3 - EmrD(–) translation and FRET kinetics evaluated by exponential fitting.

mRNA (codons)	τ_{av}[a] (s)	k_{av}[b] (aa/s)	τ_1[c] (s)	τ_2[c] (s)	τ_{post}[c]. (s)
50	23 ± 5	2.2 ± 0.4	19 ± 1	-	-
60	23 ± 3	2.6 ± 0.3	18 ± 2	-	50 ± 20
70	33 ± 6	2.1 ± 0.4	18 ± 3	40 ± 1	-
85	28 ± 5	3.1 ± 0.5	16 ± 1	-	-
110	37 ± 7	3.0 ± 0.5	19 ± 1	-	-
135	34 ± 6	4.0 ± 0.6	18 ± 3	40 ± 30	-

[a] The overall translation time (τ_{av}) is calculated from the sum of the delay time before the full-length peptide appears (τ_{delay}) and the exponential term for the synthesis of full-length peptide (τ_{exp}).

[b] k_{av} = aa chain length/τ_{av}.

[c] Results of exponential fitting of the FRET time courses to the equation that includes a delay time τ_{delay} and up to three exponential terms; $\tau_1 = \tau_{delay} + \tau_{exp1}$, $\tau_2 = \tau_{delay} + \tau_{exp2}$; $\tau_{post} = \tau_{delay} + \tau_{exp3}$.

[d] Very small signal change.
