# Supplementary figures and images for: Co‐translational insertion and topogenesis of bacterial membrane proteins monitored in real time
Source: EMBO J. 2020 Apr 20;39(15):e104054. doi: 10.15252/embj.2019104054 (PMC7396858; doi:10.15252/embj.2019104054)

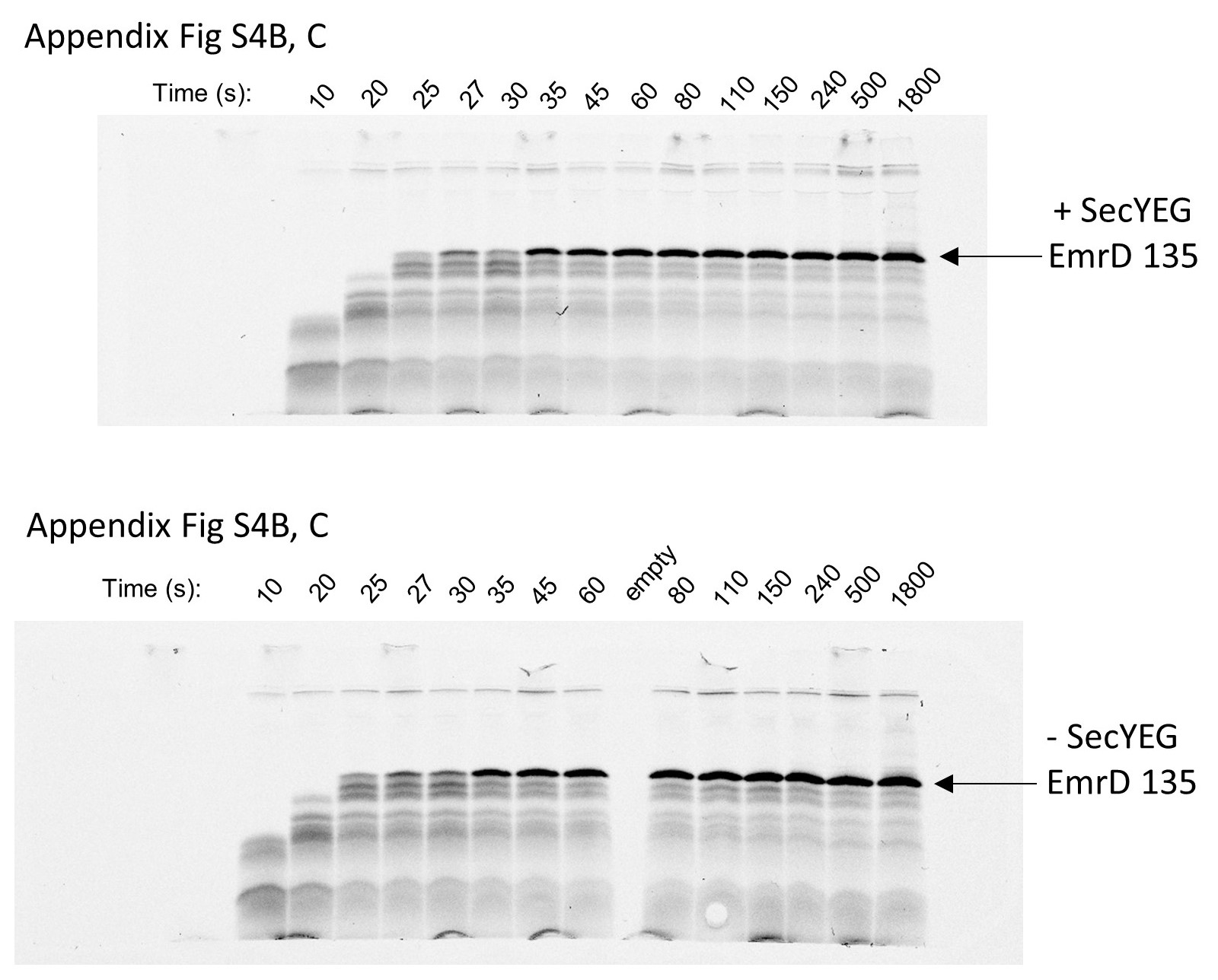

Supplement: Supplementary file 2 — Source Data for Appendix [file EMBJ-39-e104054-s003.zip › FigS4_SourceData.jpg]

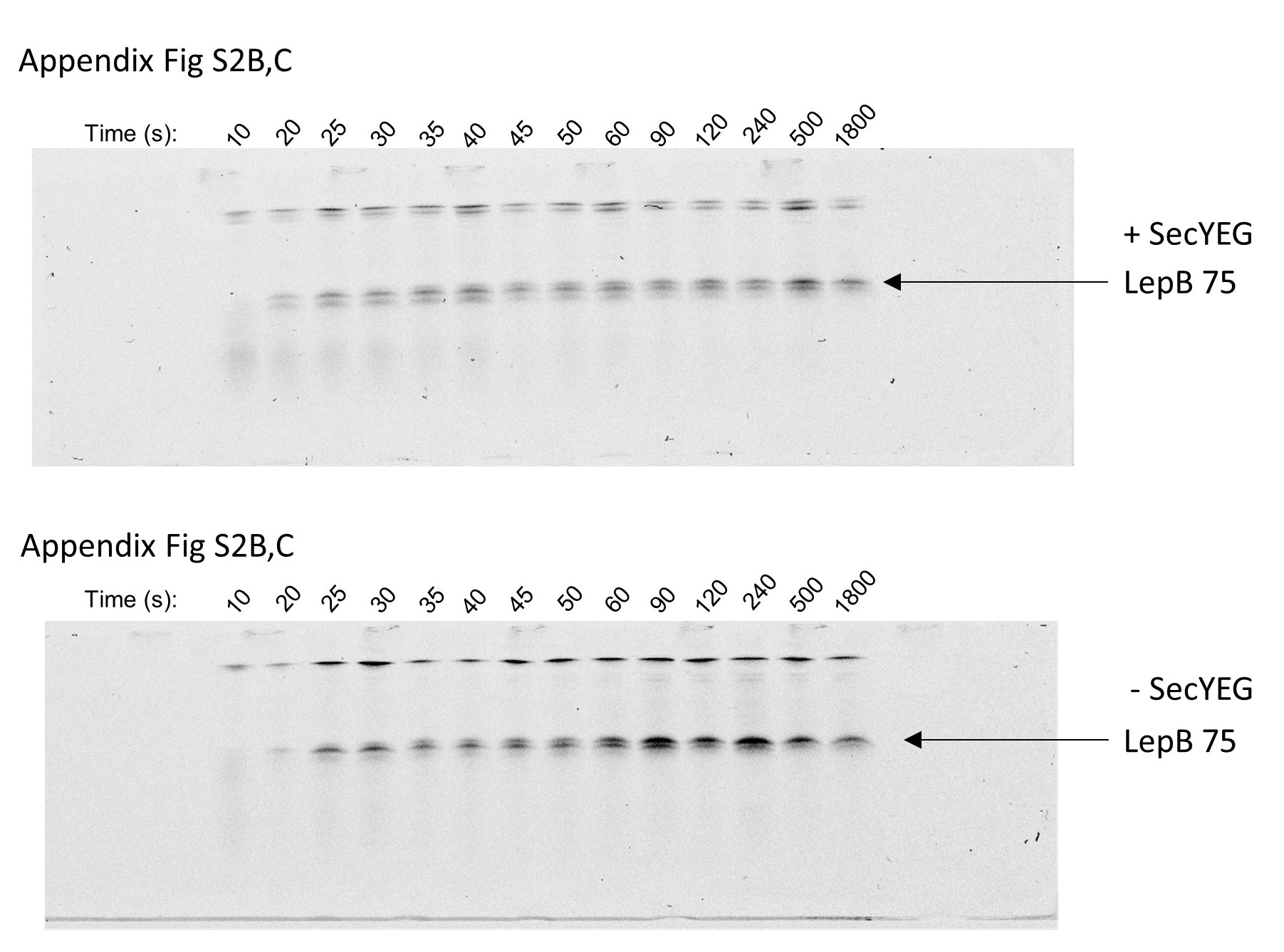

Supplement: Supplementary file 2 — Source Data for Appendix [file EMBJ-39-e104054-s003.zip › FigS2_SourceData.jpg]

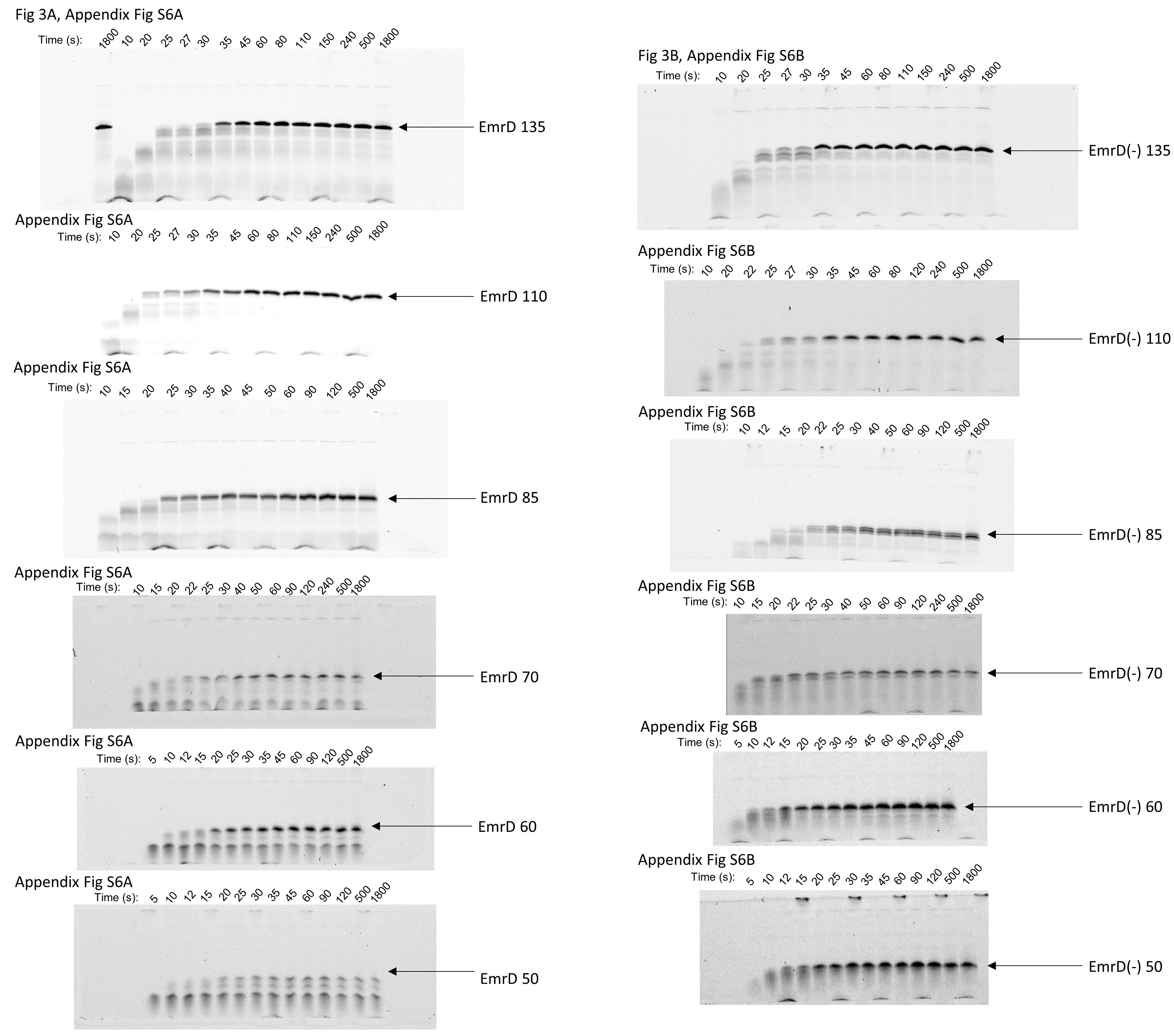

Supplement: Supplementary file 2 — Source Data for Appendix [file EMBJ-39-e104054-s003.zip › FigS6_SourceData.jpg]

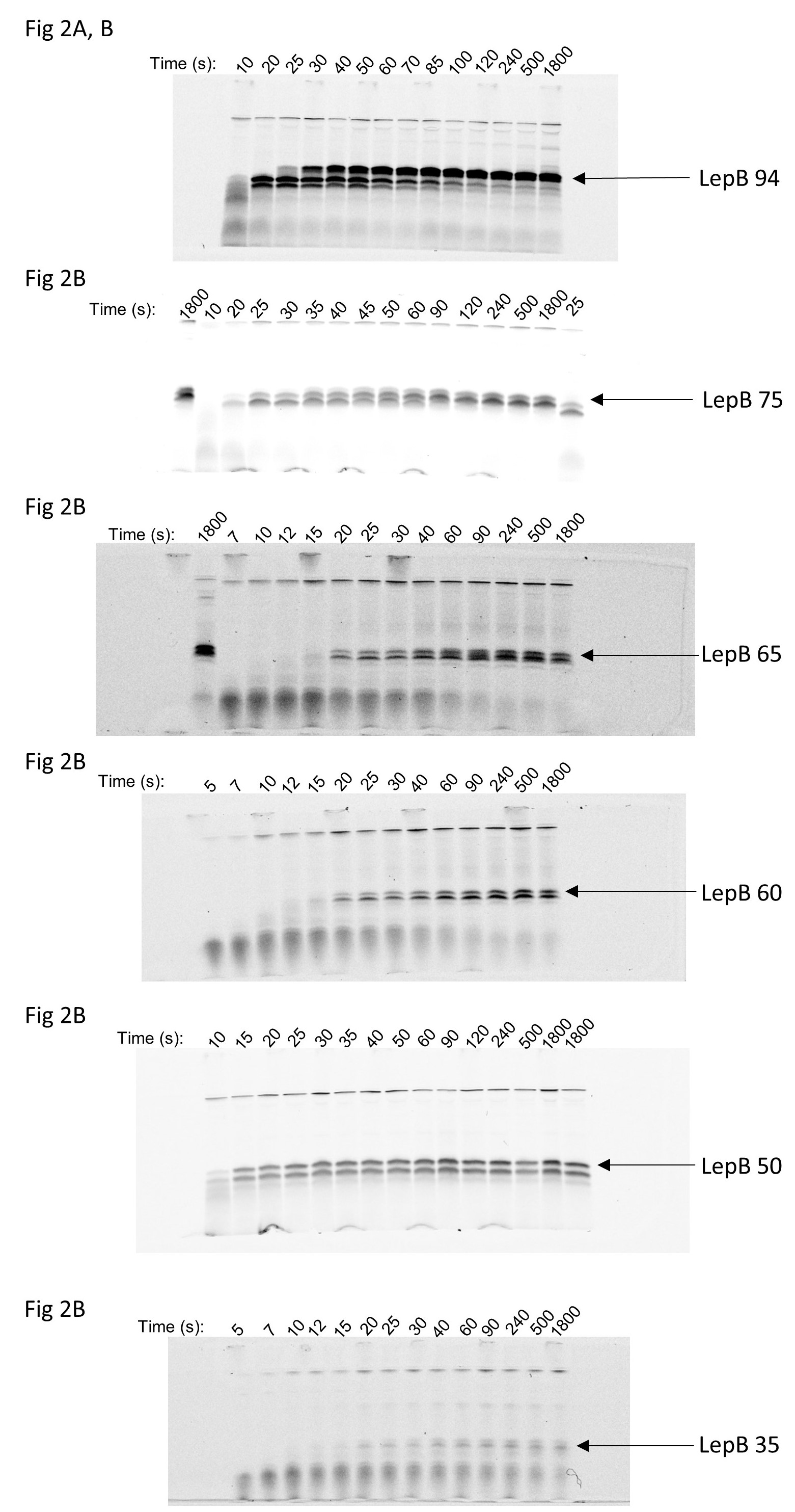

Supplement: Supplementary file 4 — Source Data for Figure 2 [file EMBJ-39-e104054-s002.jpg]
